# Unusual presentation of a giant recurrent inguinal hernia: a case report

**DOI:** 10.1093/jscr/rjaf011

**Published:** 2025-01-22

**Authors:** Senol Tahir, Frosina Jovanovska, Suad Abdiu, Maja Sofronievska Glavinov

**Affiliations:** Faculty of Medical Sciences, Goce Delcev University, Stip, North Macedonia, and University Surgery Hospital “St. Naum Ohridski”, Visceral Surgery Department, 11 Oktomvri 53, 1000 Skopje, North Macedonia; University Surgery Hospital, “St. Naum Ohridski”, Visceral Surgery Department, 11 Oktomvri 53, 1000, Skopje, North Macedonia; University Surgery Hospital “St. Naum Ohridski”, Department of Urology, 11 Oktomvri 53, 1000, Skopje, North Macedonia; University Surgery Hospital “St. Naum Ohridski”, Department of Urology, 11 Oktomvri 53, 1000, Skopje, North Macedonia; Faculty of Medical Sciences, Goce Delcev University, Stip, North Macedonia

**Keywords:** recurrent inguinal hernia, unusual presentation, case report

## Abstract

The recurrence rate of inguinal hernia is 1–10%, most often in the inguinal region, and seldom in different locations. A 72-year-old man with a large soft swelling in the right ventrolateral abdominal region without swelling in the scrotum, operated on right inguinal hernia at pediatric age. Clinical findings revealed a giant right ventrolateral hernia and abdominal CT showed weakness of the abdominal wall with a 25 cm long hernial sac with an apex under the right costal arch and a base at the deep inguinal opening, that was diagnosed as a recurrent inguinal hernia with unusual presentation. Hernioplasty without opening the hernial sac was performed in an atypical manner. The patient was discharged from the hospital without pain or discomfort at the follow-up. The common presentation of recurrent inguinal hernia is inguinal-scrotal but an unusual presentation should be reconsidered with a proper diagnosis and adequate surgical treatment.

## Introduction

The prevalence of inguinal hernia is 7.7% (6.06–9.34) with around 20 million hernia operations annually worldwide [1, 2]. Family history, gender, age, abnormal collagen metabolism, prostatectomy, low BMI, and contralateral hernia are the most frequent risk factors for inguinal hernia recurrence [2]. The recurrence rate is higher with the tension non-mash techniques (3–15%) [3], but in recent decades, with the routine use of tension-free mesh techniques, this percentage has decreased [4]. Perioperative risk factors leading to hernia recurrence are insufficient surgical technique, a small number of surgical interventions, surgical incompetence, and local anesthesia. The diagnosis of inguinal hernia (primary or recurrent) can be established by clinical examination, US, MRI, and CT [5]. Although the most common presentation of recurrent inguinal hernia is inguinal-scrotal, seldom it can have an unusual presentation as in the presented case [2, 3].

## Case report

**Figure 1:**
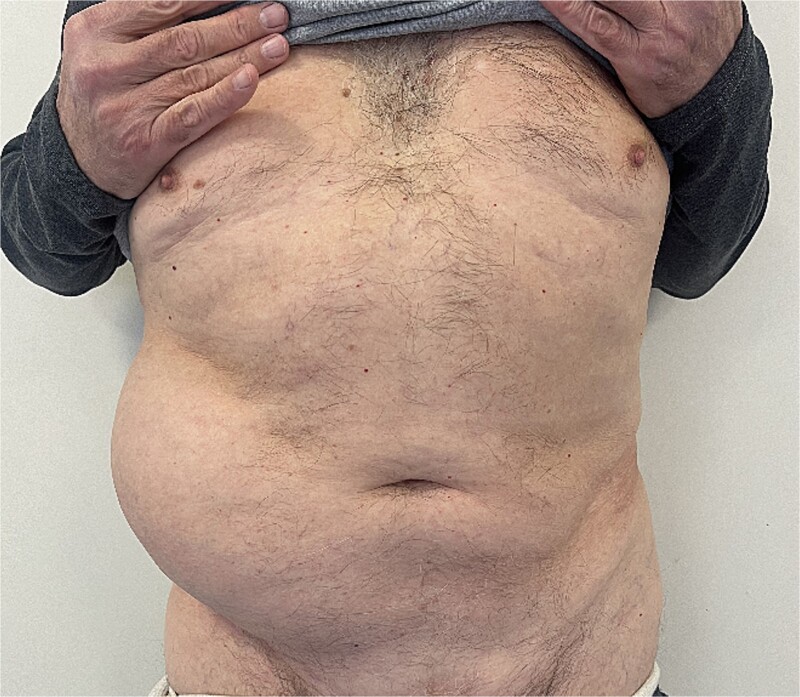
Preoperative soft mass of the right ventrolateral abdominal wall

**Figure 2:**
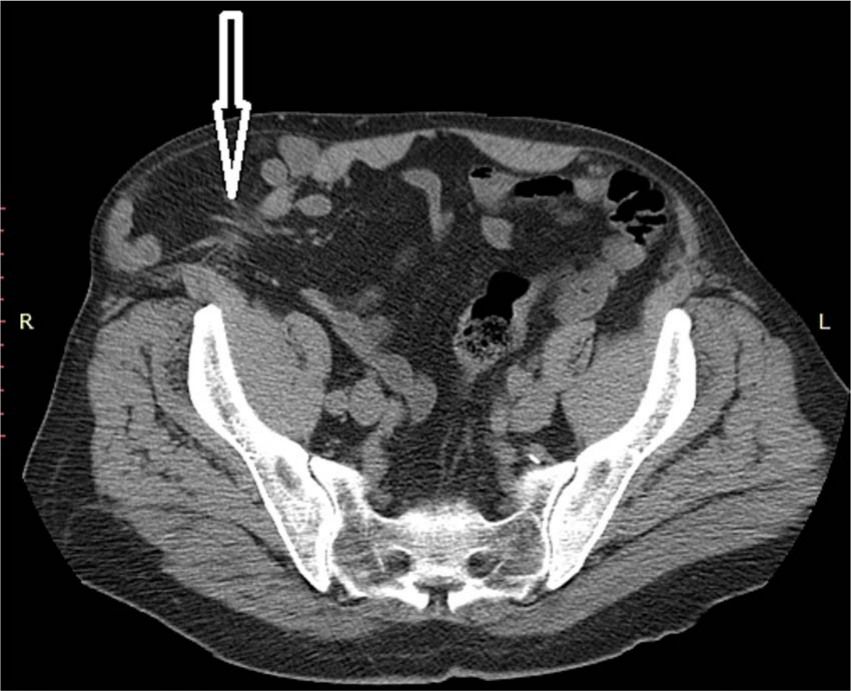
Non-contrast abdominal CT, an arrow pointing to the abdominal wall defect and intestine protrusion

**Figure 3:**
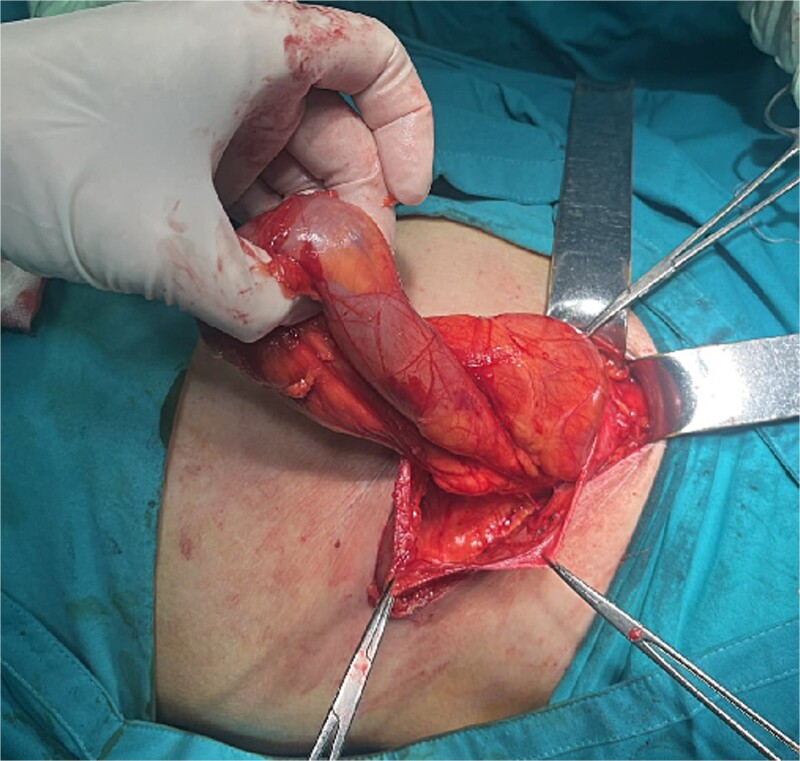
Hernia sac with small intestine loops inside that remained intact during dissection

**Figure 4:**
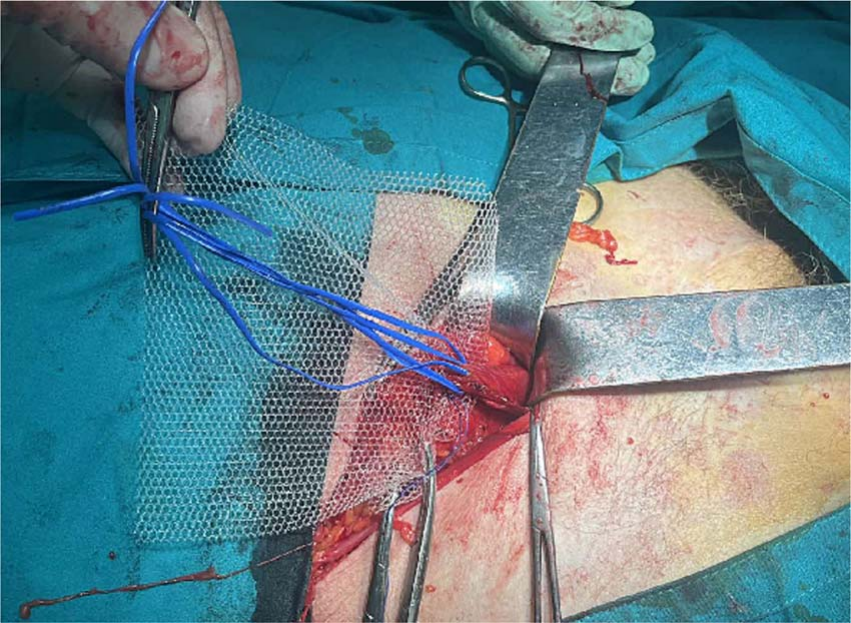
Placement of the polypropylene mesh 15 × 12 cm after dissection of the spermatic cord

**Figure 5:**
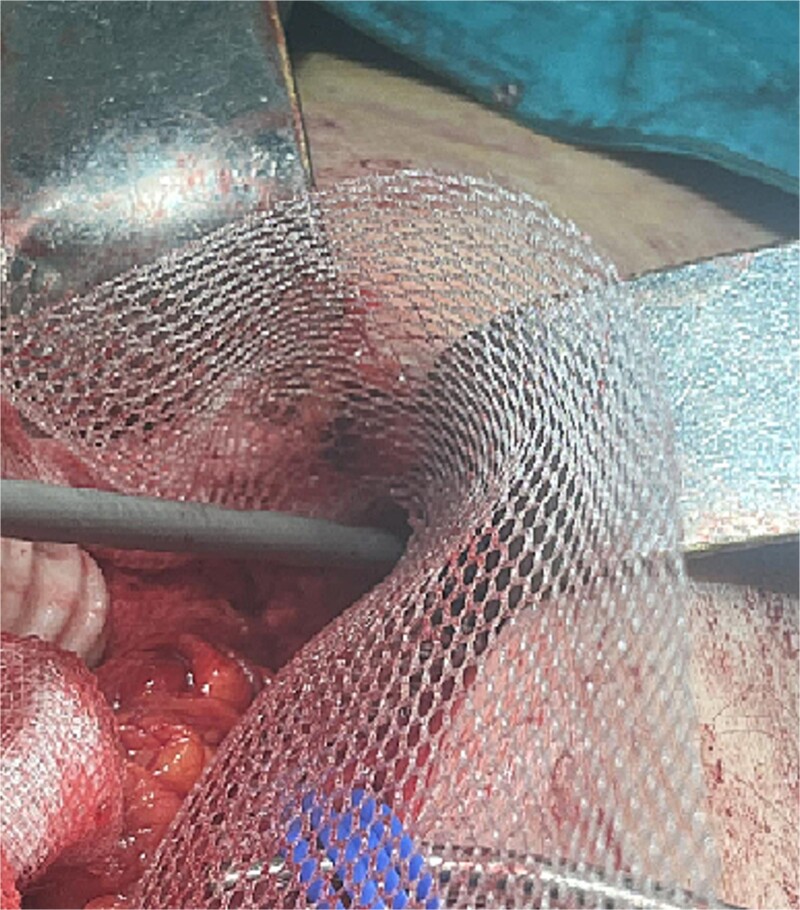
Mesh fixation to the pubic tuberculum and conjoint tendon with a laparoscopic taker

**Figure 6:**
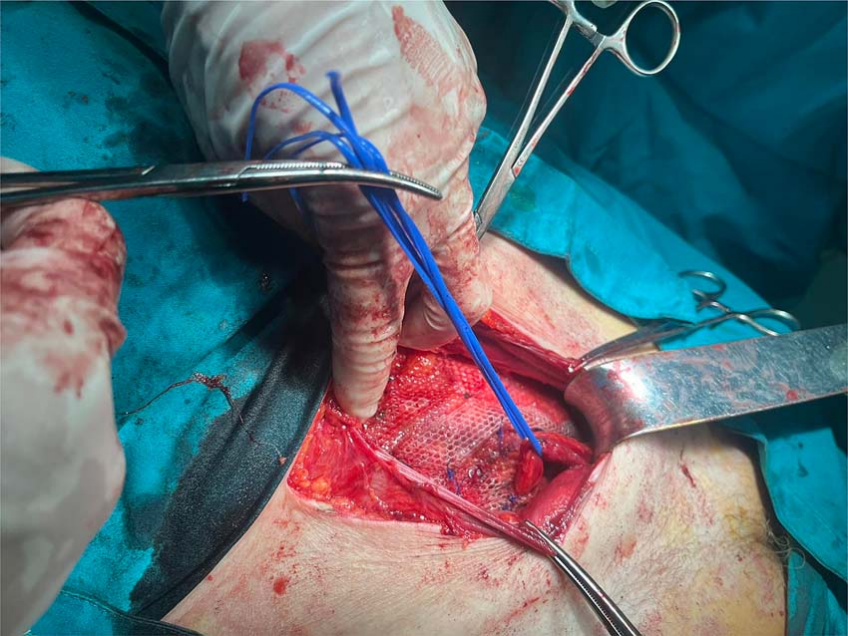
Final mesh position with the opening for the spermatic cord

**Figure 7:**
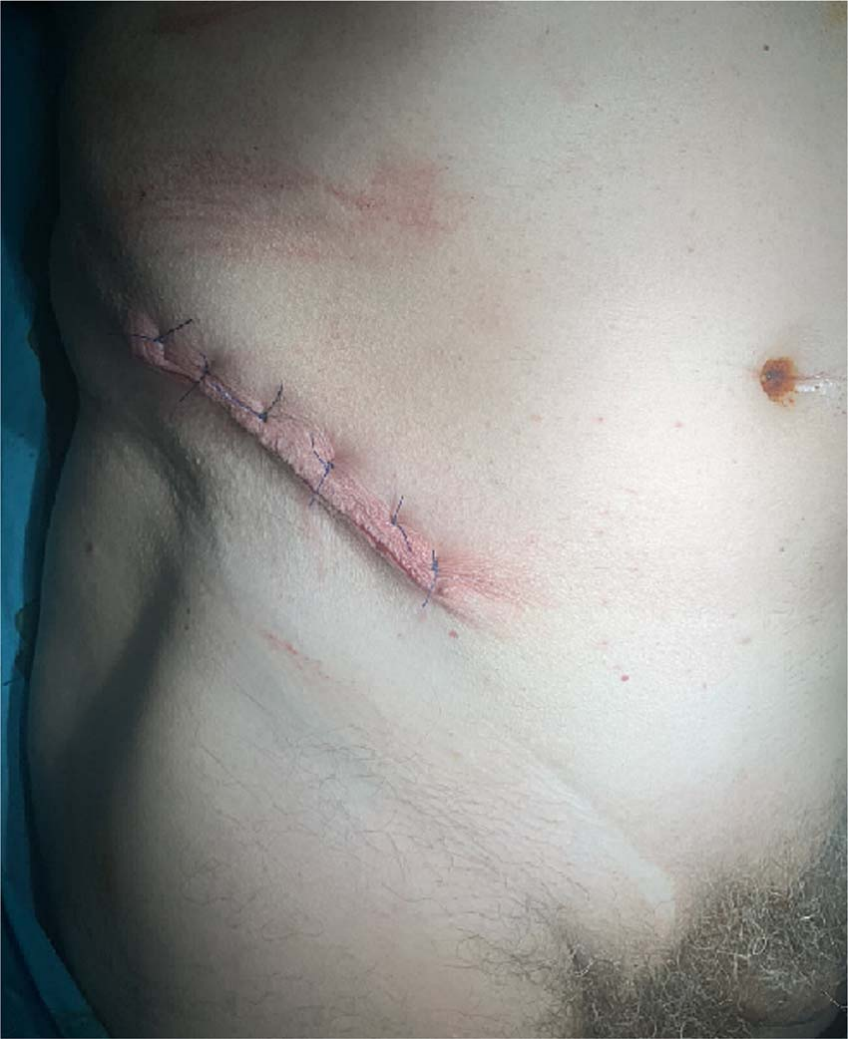
Closure of the skin incision

**Figure 8:**
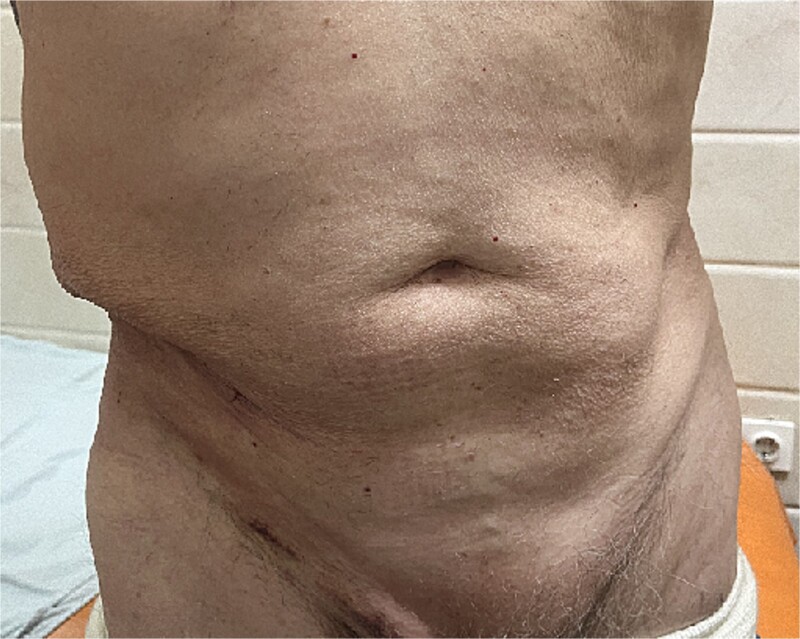
Postoperative abdominal wall restoration

A 72-year-old patient, in good physical condition with a BMI of 26.1, came for an examination due to swelling in the right lateral abdominal wall without swelling of the scrotum. The patient was operated on for a right-sided inguinal hernia as an 8-year-old boy, but there was no appropriate medical documentation for the operative technique used. A month ago, besides the swelling, he experienced occasional abdominal pain and difficulties during physical activities in his garden. During clinical examination, a soft swelling in the right lateral abdominal wall, compressible under palpation was ascertained (Fig. 1). The abdominal ultrasound and contrast abdominal CT showed a thinned right abdominal wall with superficial intestinal loops and the differential diagnosis of a large recurrent inguinal or Spigelian hernia was presumed (Fig. 2). The patient received one dose of prophylactic cephalosporin preoperatively, and was operated on under general endotracheal anesthesia, in a supine position with a lumbar pillow placed for a slight tilt to the left. A right lateral lumbar-inguinal skin incision 4–5 cm above the anterior superior iliac spine in a horizontal direction following the lumbar dermatomal lines was performed. Subcutaneous fat was gently separated and the hernial sac dissection went to its apex below the right costal arch and base to the deep inguinal opening. When the sac was completely freed, the final diagnosis of giant recurrent indirect inguinal hernia (Fig. 3), and the sac with its content was repositioned in the abdominal cavity without opening the abdominal cavity and partial closure of the internal iliac ring was performed with resorptive sutures. After the dissection of the inguinal ligament up to the pubic tubercle (without extending the skin incision) a 15 × 12 cm polypropylene mesh was placed (Fig. 4), fixed to the pubic tuberculum and conjoint tendon with a laparoscopic taker (Fig. 5), and the ileopubic ligament partly with individual and partly with continuous non-absorbable polypropylene 2/0 suture. An opening for the spermatic cord was provided and medially the mesh was fixed with individual resorptive stitches (Fig. 6). The aponeurosis of the external oblique muscle was closed as much as it allowed to be approximated followed by individual subcutaneous and skin stitches (Fig. 7). The operating time was 65 minutes without blood loss and the patient had a quick and satisfactory recovery. He was discharged from the hospital on the third post-op day, and the skin stitches were removed on the 14th day. One month after the surgical treatment the patient was in good condition without any complaints (Fig. 8).

## Discussion

This case is unique in its presentation of inguinal hernia recurrence in unusual anatomical regions and different clinical presentations since the most common presentation of recurrent inguinal hernia is inguinal-scrotal [2, 3]. The possible mechanism for such an unusual giant recurrent inguinal hernia is presumably pediatric-age surgical treatment for a right-sided inguinal hernia. Usually in these techniques, the musculoaponeurotic anatomical structures around the external hernial opening are not closed enough, and the hernial sac expands and grows toward the scrotum [6]. In the presented case, presumably a good musculo-aponeurotic closure of the inguinal canal and the external inguinal ring was performed, and the musculature closure around the deep inguinal ring became insufficient and gave rise to the peritoneum protrusion. For this reason, the hernia sac couldn’t grow medio-inferiorly towards the scrotum and spread in a lateral direction to the superior iliac spine and the lateral right lumbar region, classified as type R3 recurrent inguinal hernia [4]. The mesh technique is required following the guidelines for surgical treatment of recurrent hernias [3]. The surgeon's experience, expertise, and available resources will determine whether to use an open or laparoscopic operative technique [5]. Additionally, the patient's age, comorbidities, and health status all influence the surgical procedure decision [1].

In the presented case, the decision was made for an open approach, due to the presence of a giant hernia, with uncertainty as to whether it was a Spigelian or recurrent inguinal hernia. During the surgical procedure, all recommendations were applied: dissection and non-resection of the hernial sac, and placement of a mesh with wide openings in the appropriate size (15 × 12 cm) [7]. The mesh was placed in the desired locations (pubic tuberculum, conjoint tendon) using a laparoscopic taker to avoid skin incision extension caudally and maneuver in the small inguinal space. Resorbable sutures were used to fix the mesh above Hesselbach's triangle to prevent inguinodynia, the most frequent postoperative complication [3, 4]. Even though, drainage should be placed in giant hernias, where a greater portion of the subcutaneous or musculoaponeurotic areas is dissected [8], in the presented case, the careful and precise dissection and adequate hemostasis without any bleeding led to the decision not to put drainage. Single sutures were placed to minimize the free spaces between the subcutaneous tissue and the aponeurosis. Also, following recommendations a prophylactic single-dose cephalosporin antibiotic was given, half an hour before the start of the intervention [9]. Postoperative outcomes are good when following the surgical anatomy of the inguinal region and the surrounding anatomical regions, meticulous surgical technique, proper hemostasis, and mesh placement technique [10].

## Conclusion

The common presentation of recurrent inguinal hernia is inguinal-scrotal but an unusual presentation should be reconsidered with a proper diagnosis and adequate surgical treatment.

## Data Availability

A written consent of participation and publishing was obtained from the patient. Data is available.
